# Expressional patterns of chaperones in ten human tumor cell lines

**DOI:** 10.1186/1477-5956-2-8

**Published:** 2004-12-14

**Authors:** Jae-Kyung Myung, Leila Afjehi-Sadat, Maureen Felizardo-Cabatic, Irene Slavc, Gert Lubec

**Affiliations:** 1 Department of Pediatrics, Medical University of Vienna, Vienna, Austria

## Abstract

**Background:**

Chaperones (CH) play an important role in tumor biology but no systematic work on expressional patterns has been reported so far. The aim of the study was therefore to present an analytical method for the concomitant determination of several CH in human tumor cell lines, to generate expressional patterns in the individual cell lines and to search for tumor and non-tumor cell line specific CH expression.

Human tumor cell lines of neuroblastoma, colorectal and adenocarcinoma of the ovary, osteosarcoma, rhabdomyosarcoma, malignant melanoma, lung, cervical and breast cancer, promyelocytic leukaemia were homogenised, proteins were separated on two-dimensional gel electrophoresis with in-gel digestion of proteins and MALDI-TOF/TOF analysis was carried out for the identification of CH.

**Results:**

A series of CH was identified including the main CH groups as HSP90/HATPas_C, HSP70, Cpn60_TCP1, DnaJ, Thioredoxin, TPR, Pro_isomerase, HSP20, ERP29_C, KE2, Prefoldin, DUF704, BAG, GrpE and DcpS.

**Conclusions:**

The ten individual tumor cell lines showed different expression patterns, which are important for the design of CH studies in tumor cell lines. The results can serve as a reference map and form the basis of a concomitant determination of CH by a protein chemical rather than an immunochemical method, independent of antibody availability or specificity.

## Background

Chaperones (CH) and heat shock proteins (HSPs) play important roles in tumor biology and still are holding centre stage. The heat shock response was discovered in 1962 by Ritossa [1], who reported that elevated temperature led to the appearance of a new 'puffing' pattern in the salivary gland polytene chromosomes of *Drosophila busckii*. Since then, efforts from a large number of investigators have shown that the heat shock response is ubiquitous and highly conserved. It is observed in all organisms from bacteria to plants and animals. CH form an essential defense mechanism for protection of cells from a variety of harmful conditions, including temperature elevation or heat shock, decrease in pH, hypersalinity, alcohols, heavy metals, oxidative stress, inhibitors of energy metabolism, fever or inflammation [2,3]. This broad spectrum of functions gave rise to the term 'molecular chaperone' an entity that acts to assist other proteins' folding and maturation in the cell. However, not all HSPs are CH and not all CH are HSPs [4].

Genetic studies showed that most HSPs are essential to life. They are believed to play an indispensable role in the conformational maturation of a nascent polypeptide chain in prokaryotic and eukaryotic cells. Traditionally, HSPs are grouped into five major families according to molecular weights. They were designated HSP90 (heat shock protein of apparent molecular weight 90 kDa), HSP70 (70-kDa HSPs), HSP60 (60-kDa HSPs), HSP40 or DnaJ (40-kDa HSPs), and the small heat shock proteins (sHSPs) [5-7].

The connection of HSPs with tumor immunity was discovered in the 1980s [8-10]. It was found that structurally unaltered HSPs which are purified from tumor cells could immunize animals to generate tumor-specific immunity whereas corresponding preparations from normal tissues did not. Many recent interesting observations have been made with regards to CH's ability to regulate tumor biology. HSP70 and other CH are known to be determinants of cell death and cell transformation processes. Elevated expression of HSP70 and HSP90 in tumor cells was detected in several cases [11,12]. Recently, it has been recognised that HSPs regulate apoptosis. HSP27 and HSP70 are antiapoptotic, while HSP60 and HSP10 are proapoptotic. The ability of HSPs to protect cells from stressful stimuli suggests that these proteins play a role in tumorigenicity, with the fact that cells or tissues from various tumors have been shown to express unusually high levels of one or more HSPs. Experimental models support the role of HSPs in tumorigenesis since HSP27 and HSP70 have been shown to increase the tumorigenic potential of rodent cells in syngeneic hosts [13,14].

The contribution of HSPs to tumorigenesis may be attributed to their pleiotropic activities as molecular chaperones that provide the cancer cell with an opportunity to alter protein activities, in particular components of the cell cycle machinery, kinases and other proteins implicated in tumor progression. HSP70 chaperone activity may also influence tumorigenesis by regulating the activity of proteins that are involved in the cell cycle machinery [15].

Clinically, in a number of cancers such as leukaemia, breast cancer and endometrial cancer, an increased level of HSP27, relative to its level in non-transformed cells has been detected [16]. In addition, increased expression of HSP70 has also been reported in high-grade malignant tumors such as breast and endometrial cancer, osteosarcoma and renal cell tumors [17-19]. HSP70 levels correlate with malignancy in osteosarcoma and renal cell tumors; its expression is paradoxically associated with improved prognosis [18,20]. HSP90 and HSP60 are also over-expressed in breast tumors, lung cancer, leukaemias and Hodgkin's disease [19,21-23]. The molecular basis for over-expression of HSPs in tumors is not completely understood and may have different etiologies. For example, overexpression may be due to the suboptimal cellular environment in poorly vascularised hypoxic tumors or to growth conditions within the solid tumor [24].

Many studies have focused on the critical role of chaperones in protein folding, their relevance in protein conformational diseases and tumorigenesis. There is, however, no systematic information available on their expressional pattern in individual tumor cell lines in a wide range of tumors.

This study addresses the question of differential chaperone expression studied in ten different tumor and three normal cell lines using 2-DE and matrix-assisted laser desorption/ionization-mass spectrometry (MALDI-TOF/TOF) allowing concomitant determination of many CH at the protein chemical level rather than by immunochemical methods, independent of antibody availability and specificity.

## Results

Chaperone proteins were taken from the list of all identified proteins from ten tumor and three normal cell lines using a predetermined list of expected CH based upon our own experiments, databases and literature. Identified CH proteins made up approx. 12% of all identified proteins in all cell lines studied. All major housekeeping proteins (cytoskeleton and metabolic) expected to occur in any cell lines were present in all cell lineages studied (data not shown).

**Figure 1:**
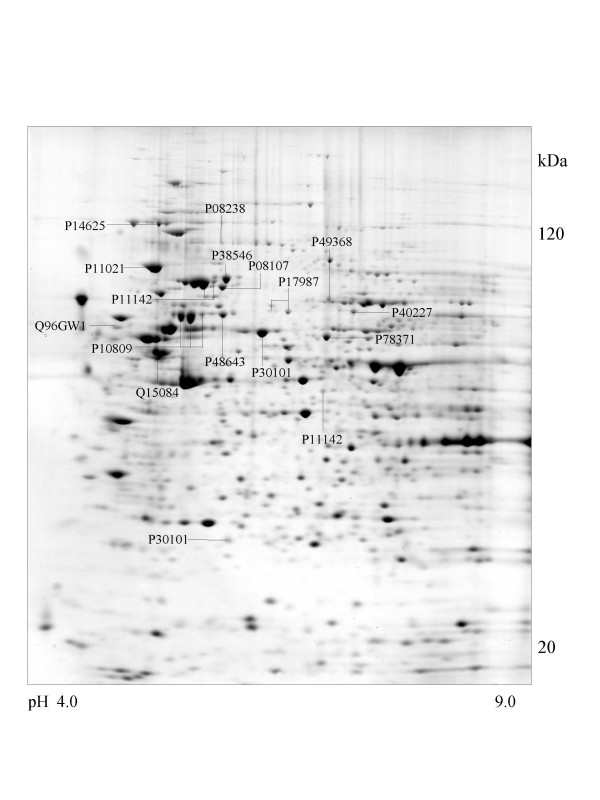
**Saos-2 cell **Colloidal Coomassie Blue stained 2D gels representing protein maps of Saos-2 cell line: Swiss prot accession numbers are used to identify proteins.

**Figure 2:**
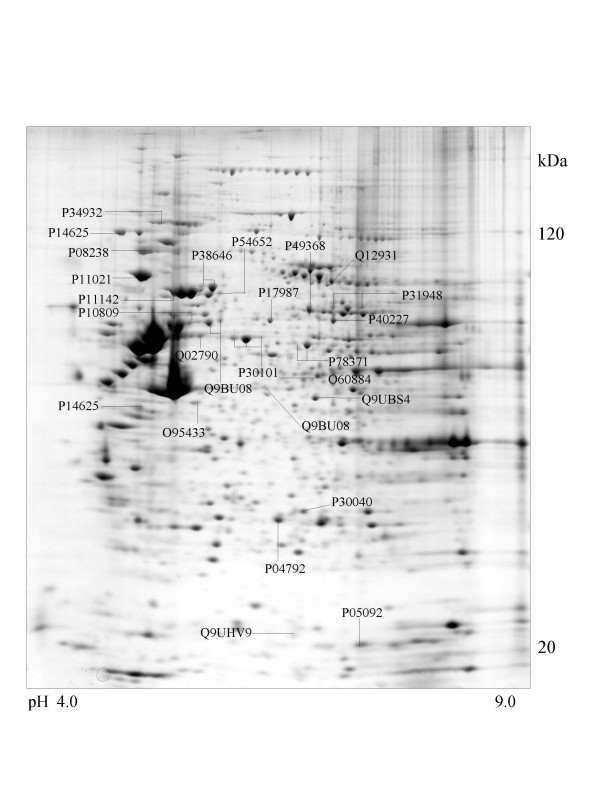
**SK-N-SH cell **Colloidal Coomassie Blue stained 2D gels representing protein maps of SK-N-SH cell line: Swiss prot accession numbers are used to identify proteins.

**Figure 3:**
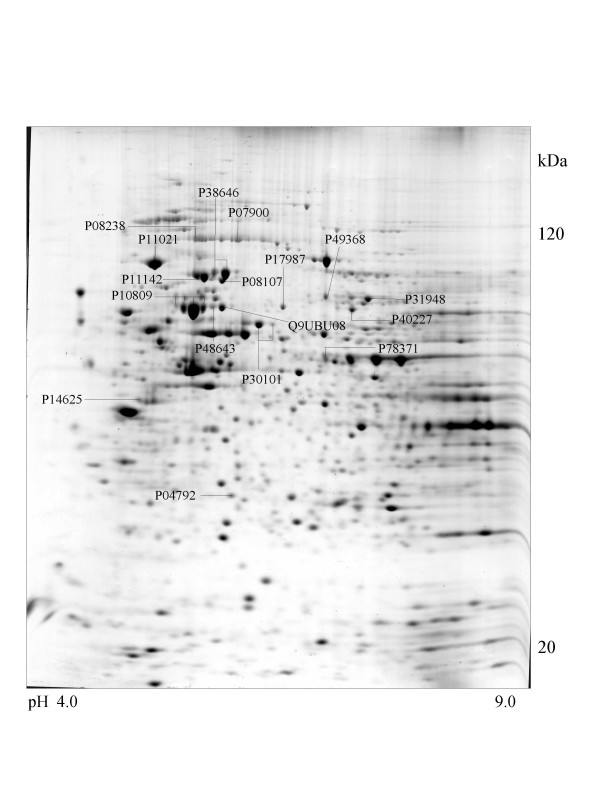
**HCT116 cell **Colloidal Coomassie Blue stained 2D gels representing protein maps of HCT 116 cell line: Swiss prot accession numbers are used to identify proteins

**Figure 4:**
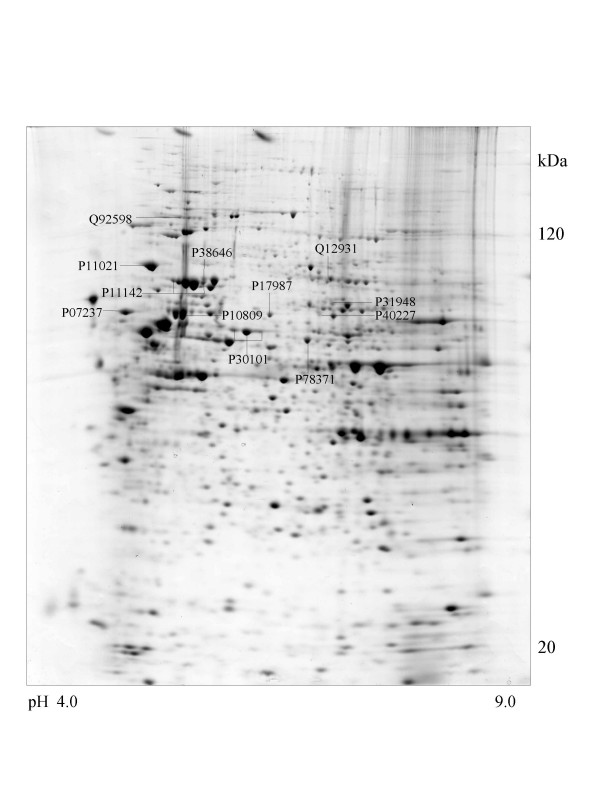
**CaOv-3 cell **Colloidal Coomassie Blue stained 2D gels representing protein maps of CaOv-3 cell line: Swiss prot accession numbers are used to identify proteins

**Figure 5:**
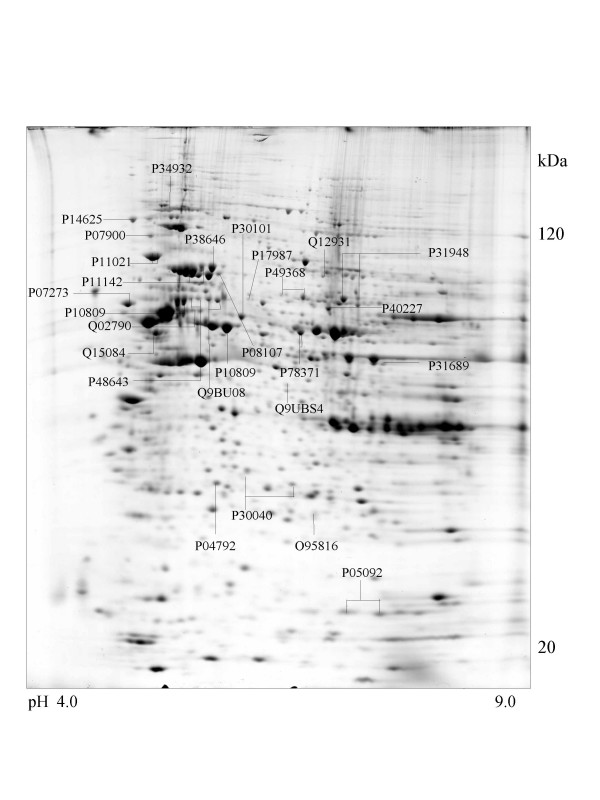
**A549 cell **Colloidal Coomassie Blue stained 2D gels representing protein maps of A549 cell line: Swiss prot accession numbers are used to identify proteins

**Figure 6:**
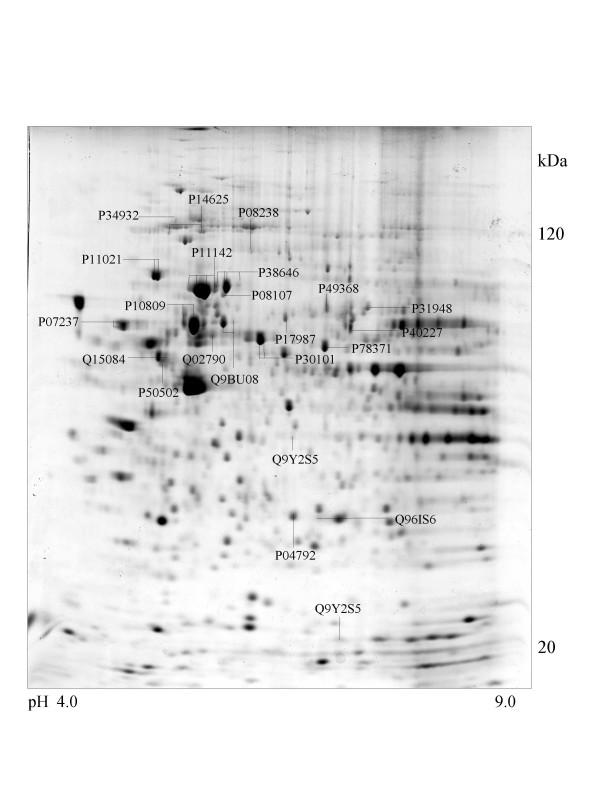
**HL-6 cell **Colloidal Coomassie Blue stained 2D gels representing protein maps of HL-6 cell line: Swiss prot accession numbers are used to identify proteins

**Figure 7:**
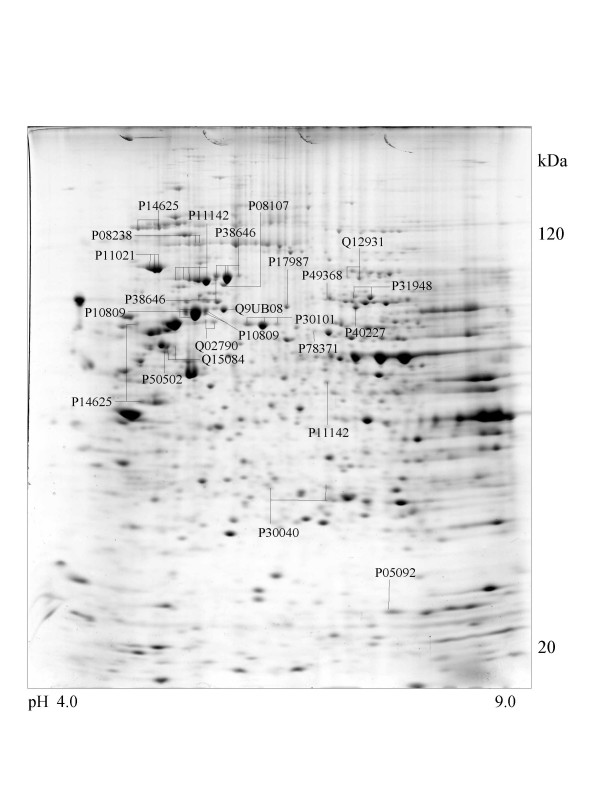
**A-375 cell **Colloidal Coomassie Blue stained 2D gels representing protein maps of A-375 cell line: Swiss prot accession numbers are used to identify proteins

**Figure 8:**
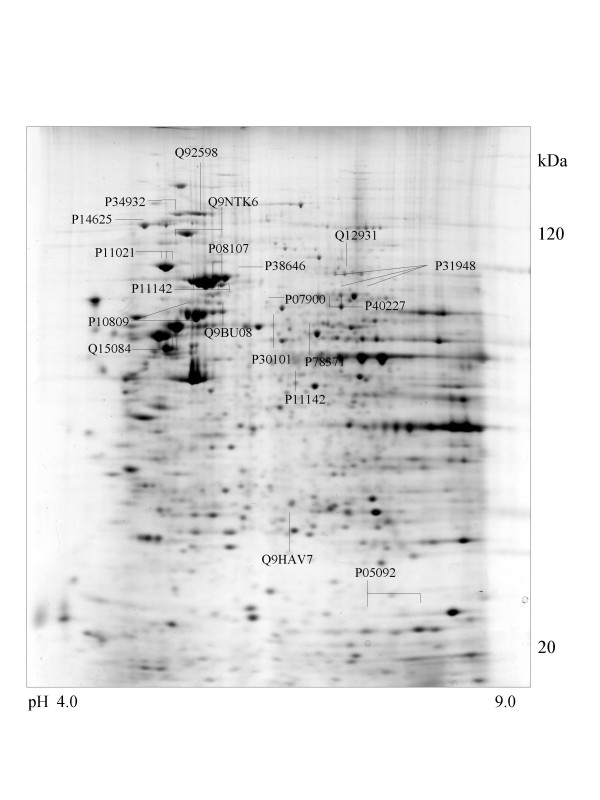
**A-673 cell **Colloidal Coomassie Blue stained 2D gels representing protein maps of A-673 cell line: Swiss prot accession numbers are used to identify proteins

**Figure 9:**
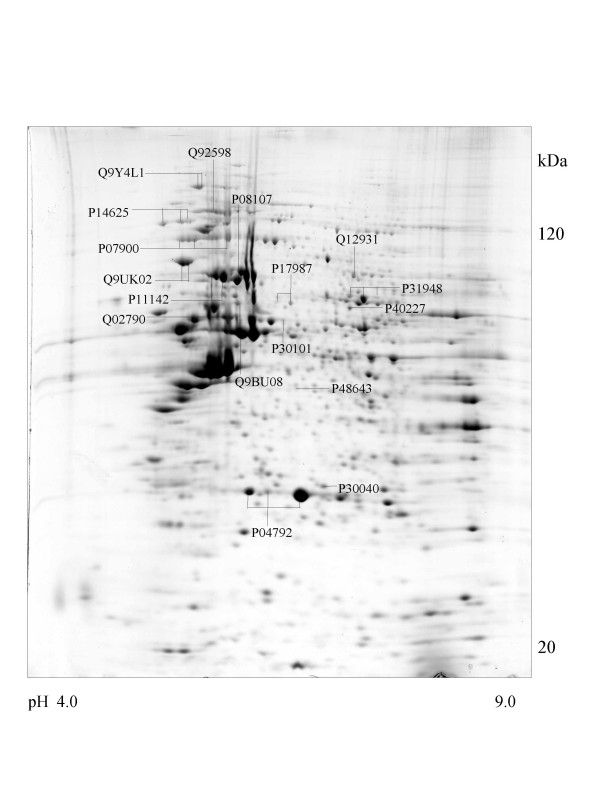
**MCF-7 cell **Colloidal Coomassie Blue stained 2D gels representing protein maps of MCF-7 cell line: Swiss prot accession numbers are used to identify proteins

**Figure 10:**
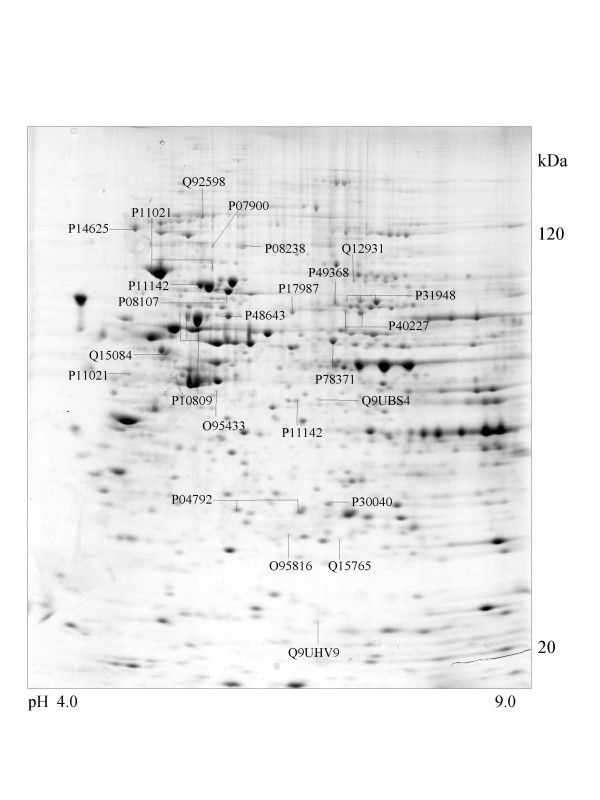
**Hela cell **Colloidal Coomassie Blue stained 2D gels representing protein maps of Hela cell line: Swiss prot accession numbers are used to identify proteins

**Figure 11:**
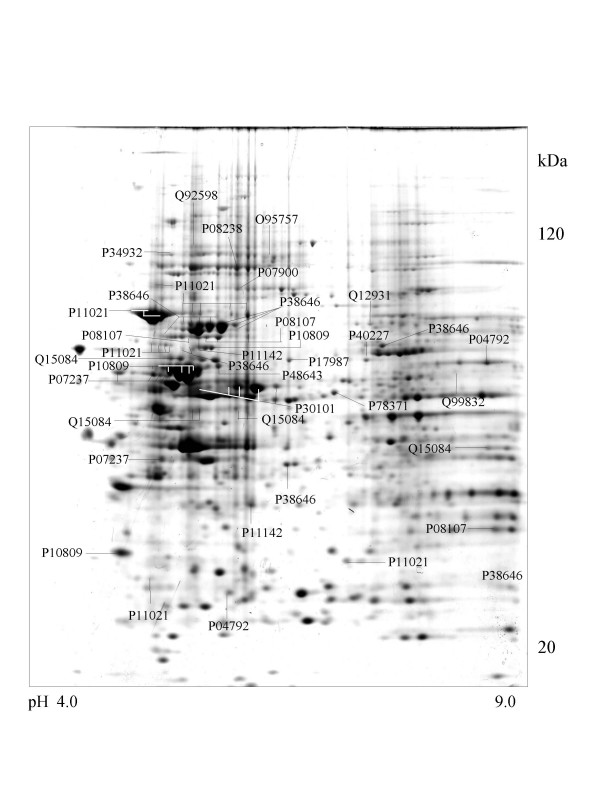
**HK-2 cell **Colloidal Coomassie Blue stained 2D gels representing protein maps of Kidney HK-2 cell line: Swiss prot accession numbers are used to identify proteins

**Figure 12:**
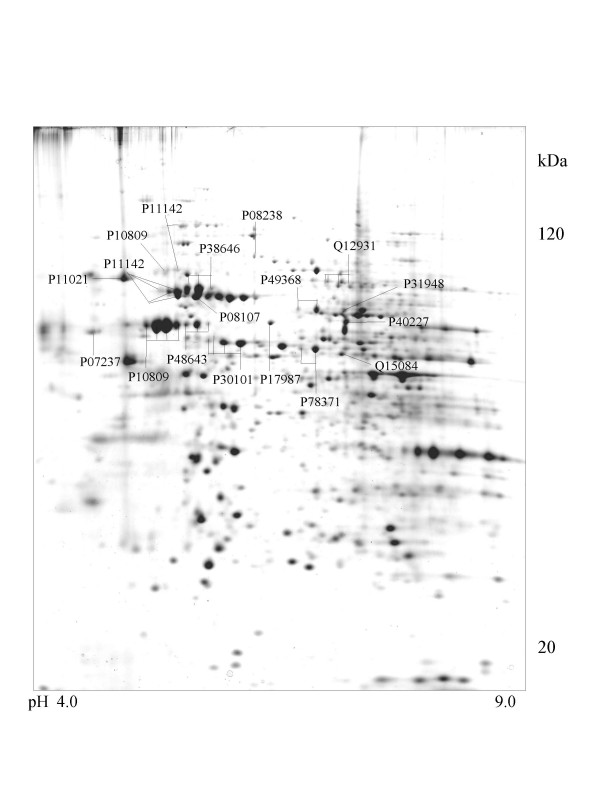
**Lymphocyte 3610 cell **Colloidal Coomassie Blue stained 2D gels representing protein maps of Lymphocyte 3610 cell line: Swiss prot accession numbers are used to identify proteins

**Figure 13:**
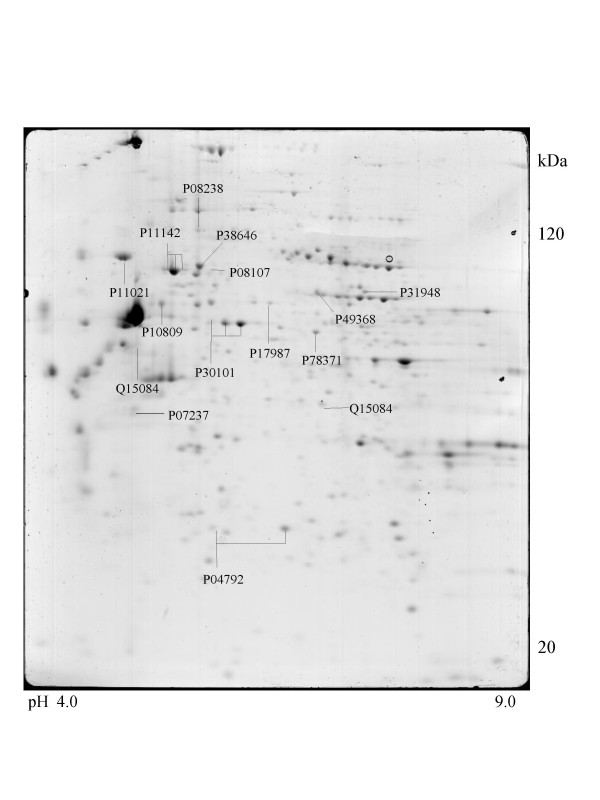
**Hs 545 SK cell **Colloidal Coomassie Blue stained 2D gels representing protein maps of Fibroblast Hs 545 SK cell line: Swiss prot accession numbers are used to identify proteins

A series of chaperone proteins with different expression patterns in ten different tumor and three normal cell lines using 2-DE and MALDI-MS were identified and listed (see Additional file 1 and 2) in Table 1 and 1-1. Chaperone proteins were classified according to their domains. Most proteins have similar p*I *values and molecular weights with theoretical value. The observed p*I *was represented in Table 2 (see Additional file 3) with the total score and the number of peptides matched. The expressional patterns of each tumor and normal cell line are shown in Figure 1,2,3,4,5,6,7,8,9,10,11,12,13.

### Proteins with HATPase_C and HSP90 domains

Prominent members of the HSP90 family of proteins are heat shock protein 90-alpha (HSP90α), heat shock protein-beta (HSP90β) and endoplasmin (GRP94) [25]. The two HSP90 isoforms are essential for the viability of eukaryotic cells. They are rather abundant constitutively, making up 1–2% of cytosolic proteins, and can be further stimulated in their expression level by stress. HSP90α and HSP90β were expressed in HCT116 and Hela cells. In addition, HSP90α was also detected in A-673, MCF-7 and A549 cell lines and HSP90β was in Saos-2, SK-N-SH, HL-60 and A375 cell lines, respectively. The isoform of HSP90β (Acc.No.Q9NTK6) was also detected in A-673 cells and is represented in Figure 8.

All cell lines except adenocarcinoma cells (CaOv-3) expressed endoplasmin (GRP94) protein that functions in the endoplasmic reticulum [26] and the protein similar to tumor rejection antigen is observed in the osteosarcoma (Saos-2) cell line represented in Figure 1.

Heat shock protein 75 kDa is named tumor necrosis factor type 1 receptor associated protein or TRAP1. The 2.4-kilobase TRAP-1 mRNA was variably expressed in skeletal muscle, liver, heart, brain, kidney, pancreas, lung, and placenta. TRAP-1 mRNA was also detected in each of eight different transformed cell lines [27]. At the protein level, it was expressed in ovary (CaOv-3), lung (A549), skin (A-375), rhabdomyosarcoma (A-673), breast cancer (MCF-7) and cervix carcinoma (Hela) cells.

### Proteins with HSP 70 domain

The HSP70 family constitutes the most conserved and best studied class of HSPs. Human cells contain several HSP70 family members including stress inducible HSP70, constitutively expressed HSC70, mitochondrial HSP75, and GRP78 localised in the endoplasmic reticulum [28]. HSP70 has been shown to increase the tumorigenicity of cancer cells in rodent models [13]. All ten tumor cells expressed HSC70 proteins and the isoform of HSC70 was also observed in promyeloblasts (HL-60).

The 105-kDa protein shows high similarity with HSP90 on peptide mapping with trypsin digestion. Except for the molecular mass, the physicochemical properties of the 105-kDa protein are similar to those of HSP90, although it has a HSP70 domain. This is detected in the brain, but not in the liver, lung, spleen, kidney, ovary, or uterus, in contrast to the wide distribution of HSP90 [29]. In case of tumor cells, it was observed in adenocarcinoma (CaOv-3), rhabdomyosarcoma (A-673), breast cancer (MCF-7) and cervix carcinoma (Hela) cells and is shown in Figure 4,8,9 and 10.

HSP70 kDa protein 1(HSP70-1) stabilises preexistent proteins against aggregation and mediate the folding of newly translated polypeptides in the cytosol as well as within organelles. HSP70-1 in mitochondria and endoplasmic reticulum plays an additional role by providing a driving force for protein translocation. They are involved in signal transduction pathways in cooperation with HSP90 and participate in all these processes through their ability to recognise non-native conformations of other proteins. Eight cell lines except SK-N-SH and CaOv-3 cells expressed HSP70-1.

Heat shock 70 kDa protein 4 was observed in SK-N-SH, HL-60 and A-673 cells and is represented in Figure 2,6 and 8.

Stress-70 protein (GRP75) was proposed to be implicated in the control of cell proliferation and cellular aging [30] and was observed in eight cell lines except MCF-7 and Hela cells.

Heat shock-related 70 kDa protein 2 and 150 kDa oxygen-regulated protein were detected in SK-N-SH and MCF-7 cells.

The best characterised GRP is a 78 kDa protein known as GRP78, which is identical to BiP, the immunoglobulin heavy chain binding protein [31]. Since GRP78 shares similar function and 60% amino acid identity with HSP70 [32], it is also categorised within the HSP70 multi-gene family [33]. GRP78 / BiP protein were expressed in all ten tumor cells.

### Proteins with Cpn60_TCP1 domain

Proteins with a Cpn60_TCP1 domain are involved in chaperonins, which belong to the 55–64 kDa family of HSP or stress proteins [34]. Mammalian HSP60, also called chaperonin, is mostly contained within the mitochondrial matrix, although it has also been detected in extramitochondrial sites. HSP60 participates in the folding of mitochondrial proteins and facilitates proteolytic degradation of misfolded or denatured proteins in an ATP-dependent manner. The chaperone function of HSP60 is regulated by HSP10, which binds to HSP60 and regulates substrate binding and ATPase activity [7]. In this study, all cells except MCF-7 expressed 60-kDa heat shock protein.

The chaperonin-containing T-complex polypeptide has many subunits. Among these, alpha, beta, gamma, epsilon and zeta units as well as the isoform of epsilon subunit were shown in 2-DE gels of tumor cell lines. T-complex protein 1, zeta subunit was detected in all ten tumor cell lines.

### Proteins with DnaJ and DnaJ_C domains

Among numerous co-chaperones for HSC70 [35], the DnaJ family is an essential group.. The human DnaJ (HSP40) family [36] is a noncanonical member of DnaJ, which lacks the zinc-finger domain. DnaJ homolog subfamily A member 1 and 2 were detected in A549 and SK-N-SH cell lines and DnaJ homolog subfamily B member 11 was found in SK-N-SH, A549 and Hela cells (Figure 2,5 and 10).

### Proteins with a thioredoxin domain

Protein disulfide isomerase, protein disulfide A3 and A6 containing 2 thioredoxin domains are members of the protein disulfide isomerase family and rearrange both intra-chain and inter-chain disulfide bonds in proteins to form the native structures. Protein disulfide isomerase was expressed in CaOv-3, A549 and HL-60 cells. Protein disulfide isomerase A3 was detected in nine cells except Hela cell and protein disulfide isomerase A6 was observed in HL-60, A-375, A-673 and Hela cell lines.

### Proteins with a TPR domain

Stress-induced-phosphoprotein 1 showing 9 TPR domains was expressed in all cell lines except Saos-2 cell. Hsc70-interacting protein with 3 TPR domains was detected in HL60 and A-375 cell. FK506-binding protein 4 has 3 TPR and 2FKBP_C domains. This protein was observed in SK-N-SH, A549, HL-60, A-375 and MCF-7 cells.

### Proteins with other domains

Heat shock 27 kDa protein (HSP27) is involved in the stabilisation of cytoskeletal proteins and in the protective mechanisms against oxidative stress by abolishing the burst of intracellular reactive oxygen species (ROS) [37]. HSP27 is the only protein detected among small heat-shock proteins in SK-N-SH, HCT, A549, HL-60, MCF-7 and Hela cell.

Peptidyl-prolyl-cis-trans isomerase A contains a Pro_isomerase domain and accelerates folding of proteins. It was observed in SK-N-SH, A549, HL-60, A-375 and A-673 cell lines.

ERP29_C has been recently characterised as a novel 29 kDa endoplasmic reticulum protein that is widely expressed in rat tissues. It plays an important role in the processing of secretory proteins within the ER [38]. SK-N-SH, A549, A-375, MCF-7 and Hela cells expressed ERP29 protein.

Prefoldin subunit 2 has a KE2 domain but profoldin subunit 3 has a prefoldin domain. Two of them were expressed in Hela cells and profoldin subunit 2 was also detected in SK-H-SH cell. The protein DUF704 is an activator of 90 kDa heat shock protein ATPase homolog 1. This protein was observed in bone marrow neuroblastoma (SK-N-SH) and Hela cells.

BAG-1 binds the ATPase domains of HSP70 and Hsc70, modulating their chaperone activity and functioning as a competitive antagonist of the co-chaperone Hip. The human BAG-1, BAG-2, and BAG-3 proteins bind with high affinity to the ATPase domain of Hsc70 and inhibit its chaperone activity. All these proteins contain a conserved 45-amino acid region near their C termini (the BAG domain) that binds Hsc70/HSP70, but they differ widely in their N-terminus [39]. This protein was observed in A549 and Hela cells.

GrpE protein homolog1 with GrpE domain and HSPC015 with DcpS domain were observed in A-673 and HL-60 cell exclusively.

Major differences between the ten tumor and the three normal cell lines were observed: 21 CH were observed in tumor cell lines only and vice versa, two CH were expressed in three normal cell lines exclusively (see Additional files 1 and 2).

## Discussion

The main outcome of the study is the generation of tumor cell specific patterns of chaperone and heat shock protein expression. The results form the basis for designing chaperone protein expression studies needed to evaluate the role of these structures in tumor biology by providing an analytical tool for the concomitant determination of CH, unambiguously identifying CH by a protein chemical rather than an immunochemical technique, independent of antibody availability and specificity.

It must be mentioned that only high abundance CH have been detected by this method and that the generated individual maps show relative abundance proteins only. A series of relatively abundant CH were presented by more than one spot indicating the presence of splicing variants or posttranslational modifications including glycosylation, phosphorylation, methylation, oxidation, truncation, to name a few and the molecular diversity is currently subject of detailed studies using advanced proteomic tools as MS-MS sequencing and Q-TOF instrumentation. Carbamylation during sample preparation may have been contributing to electrophoretic shifts as well

Methodologically, identification of proteins by MS-MS is a sound approach and the major (inherent) problem with the interpretation may be at the cell culture level: the standard protocols for cultivating the individual cell lines according to the supplier were followed, but the conditions for the individual cell cultures vary.

Different antibiotics or fungizides used may well lead to differences in CH expression and indeed, the use of geldanamycin has been already reported to induce heat shock expression in brain tissue [40]. Other tentative confounding factors as e.g. cell cycle differences, differences in growth and proliferation have to be taken into account, but it was the aim of the study to examine CH expression in cell lines in the absence of stressors or metabolic derangement, warranted by the use of standard protocols, wherefrom toxic effects are expected. Moreover, a list of non-tumor cell lines has been studied for CH expression already using this principle as well as comparable analytical technique and this may allow some comparison between tumor and non-tumor cell line CH profiles. Herein, we tested three more normal cell lines and observed a large number of differentially expressed CH between tumor and normal cell lineages. We are aware of the fact that the already reported CH expression patterns of five cell lines [41] and the three normal cell lines are not sufficient to find out differences between normal and tumor cell CH expression in general. We used herein cell types widely used in life sciences as e.g. fibroblasts, lymphocytes and kidney cells, that are representing well-characterised normal cell lines. It may be impossible to show specific differences between normal and tumor cell lines as the stem cells from which tumor cell lines originate are not generally known.

## Conclusions

Basically, differences between individual CH expression patterns may be due to different functional roles in individual cells and the presence of specific proteins in the individual cell lineages: there are CH to specifically protect individual proteins as e.g. specific chaperoning of tubulin beta 1 by the TCP complex [42] and of procollagen by HSP47 [43]. Differential CH expression may reflect or lead to tumor biological characteristics including dignity and carcinogenesis, and the method given herein provides the possibility and option to test these characteristics in a high-throughput performance. The specific nature of cell line patterns is given by the observation that only heat shock cognate 71 kDa protein and TCP zeta subunit protein were expressed non-specifically in all ten cell lines studied and previous protein expression profiles of CH in tumor cells or tissues are hereby extended and confirmed. Absence of a CH in an individual cell line could be explained by poor resolution in an individual gel but gel quality was checked by comparing general patterns and therefore this explanation is rather unlikely

The main focuses in CH protein research maybe now to investigate detection of more CHs, probably by prefractionation into different compartments [44], to characterise the splice variants expressed at the protein level and to evaluate post-translational modifications that may be responsible for a significant part of multiple expression forms.

## Methods

### Cell culture

Ten different tumor cell lines were purchased from American Type Culture Collection (ATCC). The cell lines and their ATCC numbers are given in Table 3 (see Additional file 4).

SK-N-SH (bone marrow neuroblastoma) and Hela (cervix carcinoma) cell lines were grown in Minimum Essential Medium (Eagle) with 2 mM L-glutamine and Earle's Basic Salt Solution (BSS) adjusted to contain 1.5 g/L sodium bicarbonate, 0.1 mM non-essential amino acids, and 1 mM sodium pyruvate, with 10% fetal bovine serum (FBS). The same conditions were used to culture the MCF-7 cell line except for supplementing 10% FBS with 0.01 mg/ml bovine insulin. HCT 116 (colorectal carcinoma) and Saos-2 (osteosarcoma) cell lines were cultured in McCoy's 5a medium with 90% 1.5 mM L-glutamine and 10% FBS. Ham's F12K medium with 2 mM L-glutamine adjusted to contain 1.5 g/L sodium bicarbonate and 10% FBS were used for the A549 (lung carcinoma) cell culture. HL-60 (acute promyelocytic leukaemia) cells were cultured with Iscove's modified Dulbecco's medium with 4 mM L-glutamine adjusted to contain 80% 1.5 g/L sodium bicarbonate and 20% FBS. A-673 (rhabdomyosarcoma), Caov-3 (ovarial adenocarcinoma) and A-375 (malignant melanoma) cell lines were cultured in DMEM with 4 mM L-glutamine adjusted to contain 1.5 g/L sodium bicarbonate and 4.5 g/L glucose with 10% FBS.

Three normal cell lines, fibroblasts, lymphocytes and kidney cells were used:

Fibroblasts, Hs 545 SK ATCC CRL-7318 (Manassas, VA), were obtained from human skin and grown in monolayers in Dulbecco's modified Eagle's medium (DMEM) containing 4 mM L-glutamine adjusted to contain 1.5 g/L sodium bicarbonate, 4.5 g/L glucose, with 10% fetal bovine serum (FBS), Penicillin and Streptomycin (GIBCO BRL) according to standard techniques Lymphocyte cell line 3610 is a spontaneously EBV transformed cell line from a patient with osteosarcoma and was obtained from the St. Anna Kinderspital-Forschungsinstitut (Vienna, Austria). The cell line was established from peripheral heparinized blood by a density gradient centrifugation using Ficoll-Paque (AMERSHAM BIOSCIENCE, Uppsala, Sweden) and grown in RPMI 1640 with 10% FBS, 70 μM gentamicin sulfate and 2 mM glutamine at a density of 2 × 10^6 ^cells per ml in 96 well plates.

HK-2 cells (Human Kidney 2) are proximal tubular epithelial cells derived from normal kidney and were purchased from ATCC CRL-2190. Cells were cultured in Keratinocyte-Serum Free Medium (GIBCO-BRL 17005–042) with 5 ng/ml recombinant epidermal growth factor (positive for alkaline phosphatase, gamma glutamyltranspeptidase, leucine aminopeptidase, acid phosphatase, cytokeratin, alpha 3 beta 1 integrin, fibronectin; negative for factor VIII-related antigen, 6.19 antigen and CALLA endopeptidase) and 0.05 mg/ml bovine pituitary extract.

All cell cultures were maintained in a humified atmosphere of 5% (v/v) CO_2 _in air at 37°C and logarithmically growing cells were harvested by trypsinisation.

### Sample preparation

Harvested cells were washed three times with 10 mL PBS (phosphate buffered saline) (Gibco BRL, Gaithersburg, MD, USA) and centrifuged for 10 min at 800 g at room temperature. The supernatant was discarded and the pellet was suspended in 1 ml of sample buffer consisting of 40 mM Tris, 7 M urea (Merck, Darmstadt, Germany), 2 M thiourea (Sigma, St. Louis, MO, USA), 4% CHAPS (3-[(3-cholamidopropyl) dimethylammonio]-1-propane-sulfonate) (Sigma, St. Louis, MO, USA), 65 mM 1,4-dithioerythritol (Merck, Germany), 1 mM EDTA (ethylenediaminetetraacetic acid) (Merck, Germany), protease inhibitors complete (Roche, Basel, Switzerland) and 1 mM phenylmethylsulfonyl fluoride (PMSF). The suspension was sonicated for approximately 30 sec in an ice bath. After homogenisation samples were left at room temperature for 1 h and centrifuged at 14,000 rpm for 1 h. The supernatant was transferred into Ultrafree-4 centrifugal filter units (Millipore, Bedford, MA), for desalting and concentrating proteins. Protein content of the supernatant was quantified by the Bradford protein assay system [45]. The standard curve was generated using bovine serum albumin and absorbance was measured at 595 nm.

### Two-dimensional gel electrophoresis (2-DE)

Samples prepared from each cell line were subjected to 2-DE as described elsewhere [46]. 1 mg protein was applied on immobilised pH 3–10 nonlinear gradient strips in sample cups at their basic and acidic ends. Focusing was started at 200 V and the voltage was gradually increased to 5000 V at a rate of 3 V/min and then kept constant for a further 24 h (approximately 180,000 Vhs totally). After the first dimension strips (18 cm) were equilibrated for 15 min in a buffer containing 6 M urea, 20% glycerol, 2% SDS, 2% DTT and then for 15 min in the same buffer containing 2.5% iodoacetamide instead of DTT. After equilibration, strips were loaded on 9–16% gradient sodium dodecylsulfate polyacrylamide gels for second-dimensional separation. Gels (180 × 200 × 1.5 mm) were run at 40 mA per gel. Immediately after the second dimension run, gels were fixed for 18 h in 50% methanol, containing 10% acetic acid, the gels were stained with Colloidal Coomassie Blue (Novex, San Diego, CA) for 12 h on a rocking shaker. Molecular masses were determined by running standard protein markers (Biorad Laboratories, Hercules, CA) covering the range 10–250 kDa. p*I *values were used as given by the supplier of the immobilised pH gradient strips (Amersham Bioscience, Uppsala, Sweden). Excess of dye was washed out from the gels with distilled water and gels were scanned with an ImageScanner (Amersham Bioscience). Electronic images of the gels were recorded using Adobe Photoshop and Microsoft Power Point softwares.

### Matrix-assisted laser desorption ionisation mass spectrometry (MALDI-MS)

Spots visualised by Colloidal Coomassie Blue staining were excised with a spot picker (PROTEINEER sp™, Bruker Daltonics, Germany), placed into 96-well microtiter plates and in-gel digestion and sample preparation for MALDI analysis were performed by an automated procedure (PROTEINEER dp™, Bruker Daltonics) [44,47]. Briefly, all visible spots were excised and washed with 10 mM ammonium bicarbonate and 50% acetonitrile in 10 mM ammonium bicarbonate. After washing, gel plugs were shrunk by addition of acetonitrile and dried by blowing out the liquid through the pierced well bottom. The dried gel pieces were reswollen with 40 ng/μl trypsin (Roche Diagnostics, Penzberg, Germany) in enzyme buffer (consisting of 5 mM Octyl β-D-glucopyranoside (OGP) and 10 mM ammonium bicarbonate) and incubated for 4 h at 30°C. Peptide extraction was performed with 10 μl of 1% TFA in 5 mM OGP. Extracted peptides were directly applied onto a target (AnchorChip™, Bruker Daltonics) that was load with α-cyano-4-hydroxy-cinnamic acid (CHCA) (Bruker Daltonics) matrix thinlayer. The mass spectrometer used in this work was an Ultraflex™ TOF/TOF (Bruker Daltonics) operated in the reflector for MALDI-TOF peptide mass fingerprint (PMF) or LIFT mode for MALDI-TOF/TOF with a fully automated mode using the FlexControl™ software. An accelerating voltage of 25 kV was used for PMF. Calibration of the instrument was performed externally with [M+H]^+ ^ions of angiotensin I, angiotensin II, substance P, bombesin, and adrenocorticotropic hormones (clip 1–17 and clip 18–39). Each spectrum was produced by accumulating data from 200 consecutive laser shots. Those samples which were analysed by PMF from MALDI-TOF were additionally analysed using LIFT-TOF/TOF MS/MS from the same target. A maximum of three precursor ions per sample were chosen for MS/MS analysis. In the TOF1 stage, all ions were accelerated to 8 kV under conditions promoting metastable fragmentation. After selection of jointly migrating parent and fragment ions in a timed ion gate, ions were lifted by 19 kV to high potential energy in the LIFT cell. After further acceleration of the fragment ions in the second ion source, their masses could be simultaneously analysed in the reflector with high sensitivity. PMF and LIFT spectra were interpreted with the Mascot software (Matrix Science Ltd, London, UK). Database searches, through Mascot, using combined PMF and MS/MS datasets were performed via BioTools 2.2 software (Bruker). A mass tolerance of 100 ppm and 1 missing cleavage site for PMF and MS/MS tolerance of 0.5 Da and 1 missing cleavage site for MS/MS search were allowed and oxidation of methionine residues was considered. The probability score calculated by the software was used as criterion for correct identification.

The algorithm used for determining the probability of a false positive match with a given mass spectrum is described elsewhere [48].

## List of abbreviations

CH chaperone

HSP heat shock protein

2-DE two dimensional gel electrophoresis

MALDI-MS matrix-assisted laser desorption ionisation mass spectrometry

OGP octyl-β-D-glucopyranoside

CHCA α-cyano-4-hydroxy-cinnamic acid

PMF peptide mass fingerprint

GRP glucose regulated protein

TCP t-complex protein

## Competing interests

The author(s) declare that they have no competing interests.

## Authors contributions

JKM did data mining and contributed to the preparation of the manuscript. LAS performed protein extraction, two-dimensional electrophoresis and data handling. MFC carried out MALDI-TOF-TOF analyses. IS developed methodology for studying proteins from cell lines. GL initiated and planned the study developing the concept, supervised 2-DE, mass spectrometry, creating data and the manuscript. All authors have read and approved the final manuscript.

## Supplementary Material

Additional File 1 Table 1. Identified proteins in different human tumor cell lines: Saos-2, SK-N-SH, HCT 116, CaOv-3, A549, HL-60, A-375, A-673, MCF-7 and Hela. Click here for file

Additional File 2 Table 1-1. Identified proteins in different normal cell lines: Kidney, Lymphocyte, Fibroblast Click here for file

Additional File 3 Table 2. Theoretical molecular weight, theoretical p*I*, observed p*I*, total score and peptide matched of molecular chaperones in tumor cell lines Click here for file

Additional File 4 Table 3. The list of tumor cell lines Click here for file
