# Supplementary material for: Expressional patterns of chaperones in ten human tumor cell lines
Source: Proteome Sci. 2004 Dec 14;2:8. doi: 10.1186/1477-5956-2-8 (PMC543454; doi:10.1186/1477-5956-2-8)
Supplement: Additional File 1 — Table 1. Identified proteins in different human tumor cell lines: Saos-2, SK-N-SH, HCT 116, CaOv-3, A549, HL-60, A-375, A-673, MCF-7 and Hela. [file 1477-5956-2-8-S1.doc]

**Table 1.** Identified proteins in different human tumor cell lines: Saos-2^1^, SK-N-SH^2^, HCT 116^3^, CaOv-3^4^, A549^5^, HL-60^6^, A-375^7^, A-673^8^, MCF-7^9^ and Hela^10^ (+ : Detected, - : Not-Detected, Acc. No: Accession Number in Swissprot, Abbr. Name: Abbreviation Name)

| **Acc. No** | **Abbr. Name** | Protein Name | **Domain** | Saos-2 | **SK-N-SH** | HCT 116 | **CaOv-3** | **A549** | **HL-60** | **A-375** | **A-673** | **MCF-7** | **Hela** |
| --- | --- | --- | --- | --- | --- | --- | --- | --- | --- | --- | --- | --- | --- |
| HSP90 / HATPas_C | | |  |  |  |  |  |  |  |  |  |  |  |
| P07900 | HS9A_HUMAN | Heat shock protein HSP 90-alpha | HATPas_C  HSP90 | - | - | + | - | + | - | - | + | + | + |
| P08238 | HS9B_HUMAN | Heat shock protein HSP 90-beta | HATPas_C  HSP90 | + | + | + | - | - | + | + | - | - | + |
| P14625 | ENPL_HUMAN | Endoplasmin [Precursor] | HATPas_C  HSP90 | + | + | + | - | + | + | + | + | + | + |
| Q12931 | TRAL_HUMAN | Heat shock protein 75 kDa, mitochondrial [Precursor] | HATPas_C  HSP90 | - | + | - | + | + | - | + | + | + | + |
| Q9NTK6 | Q9NTK6 | 88.1% homologous to isoform of HSP90-beta | HATPas_C  HSP90 | - | - | - | - | - | - | - | + | - | - |
| Q96GW1 | Q96GW1 | Similar to tumor rejection antigen (gp96) 1 | HATPase_C | + | - | - | - | - | - | - | - | - | - |
| **HSP70** |  |  |  |  |  |  |  |  |  |  |  |  |  |
| Q92598 | H105_HUMAN | Heat-shock protein 105 kDa | HSP70 | - | - | - | + | - | - | - | + | + | + |
| P08107 | HS71_HUMAN | Heat shock 70 kDa protein 1 | HSP70 | + | - | + | - | + | + | + | + | + | + |
| P54652 | HS72_HUMAN | Heat shock-related 70 kDa protein 2 | HSP70 | - | + | - | - | - | - | - | - | - | - |
| P34932 | HS74_HUMAN | Heat shock 70 kDa protein 4 | HSP70 | - | + | - | - | + | + | - | + | - | - |
| P38646 | GR75_HUMAN | Stress-70 protein, mitochondrial [Precursor] | HSP70 | + | + | + | + | + | + | + | + | - | - |
| P11021 | GR78_HUMAN | 78 kDa glucose-regulated protein [Precursor] | HSP70 | + | + | + | + | + | + | + | + | - | + |
| Table 1. Continued | | |  |  |  |  |  |  |  |  |  |  |  |
| **Acc. No** | **Abbr. Name** | Protein Name | Domain | **Saos-2** | **SK-N-SH** | HCT 116 | **CaOv-3** | **A549** | **HL-60** | **A-375** | **A-673** | **MCF-7** | **Hela** |
| **HSP70- continued** | | |  |  |  |  |  |  |  |  |  |  |  |
| Q9UK02 | Q9UK02 | BiP protein [Fragment] | HSP70 | - | - | - | - | - | - | - | - | + | - |
| P11142 | HS7C_ HUMAN | Heat shock cognate 71 kDa protein | HSP70 | + | + | + | + | + | + | + | + | + | + |
| Q9Y4L1 | OXRP_HUMAN | 150 kDa oxygen-regulated protein [Precursor] | HSP70 | - | - | - | - | - | - | - | - | + | - |
| Q96IS6 | Q96IS6 | Similar to heat shock cognate 71-kd protein | HSP70 | - | - | - | - | - | + | - | - | - | - |
| **Cpn60_TCP1** | | |  |  |  |  |  |  |  |  |  |  |  |
| P10809 | CH60_HUMAN | 60 kDa heat shock protein, mitochondrial [Precursor] | Cpn60_TCP1 | + | + | + | + | + | + | + | + | - | + |
| P17987 | TCPA_HUMAN | T-complex protein 1, alpha subunit | Cpn60_TCP1 | + | + | + | + | + | + | + | **-** | + | + |
| P78371 | TCPB_HUMAN | T-complex protein 1, beta subunit | Cpn60_TCP1 | + | + | + | + | + | + | + | + | - | + |
| P49368 | TCPG_HUMAN | T-complex protein 1, gamma subunit | Cpn60_TCP1 | + | + | + | - | + | + | + | - | - | + |
| P48643 | TCPE_HUMAN | T-complex protein 1, epsilon subunit | Cpn60_TCP1 | + | - | + | - | + | - | - | - | + | + |
| P40227 | TCPZ_HUMAN | T-complex protein 1, zeta subunit | Cpn60_TCP1 | + | + | + | + | + | + | + | + | + | + |
| Q9BU08 | Q9BU08 | 96% homologous to isororm of T-complex protein 1, epsilon subunit | Cpn60_TCP1 | - | + | + | - | + | + | + | + | + | - |
| **DnaJ** |  |  |  |  |  |  |  |  |  |  |  |  |  |
| P31689 | DJA1_HUMAN | DnaJ homolog subfamily A member1 | DnaJ | - | - | - | - | + | - | - | - | - | - |
| O60884 | DJA2_HUMAN | DnaJ homolog subfamily A member 2 | DnaJ  (1J, 1CRdomain) | - | + | - | - | - | - | - | - | - | - |
| Q9UBS4 | DJBB_HUMAN | DnaJ homolog subfamily B member 11 [Precursor] | DnaJ  DnaJ_C | - | + | - | - | + | - | - | - | - | + |
| Table 1. Continued | | |  |  |  |  |  |  |  |  |  |  |  |
| **Acc. No** | **Abbr. Name** | Protein Name | Domain | **Saos-2** | **SK-N-SH** | HCT 116 | **CaOv-3** | **A549** | **HL-60** | **A-375** | **A-673** | **MCF-7** | **Hela** |
| **Thioredoxin** | | |  |  |  |  |  |  |  |  |  |  |  |
| P07237 | PDI_HUMAN | Protein disulfide isomerase[precursor] | 2Thioredoxin | - | - | - | + | + | + | - | - | - | - |
| P30101 | PDA3_HUMAN | Protein disulfide  isomerase A3 [Precursor] | 2Thioredoxin | + | + | + | + | + | + | + | + | + | - |
| Q15084 | PDA6_HUMAN | Protein disulfide somerase A6 [Precursor] | 2 Thioredoxin | + | - | - | - | + | + | + | + | - | + |
| **Pro_isomerase** | |  |  |  |  |  |  |  |  |  |  |  |  |
| P05092 | PPIA_HUMAN | Peptidyl-prolyl cis-trans isomerase A | Pro_isomerase | - | + | - | - | + | + | + | + | - | - |
| **TPR** |  |  |  |  |  |  |  |  |  |  |  |  |  |
| P31948 | IEFS_HUMAN | Stress-induced-phosphoprotein 1 | 9TPR | - | + | + | + | + | + | + | + | + | + |
| P50502 | ST13_HUMAN | Hsc70-interacting protein | 3TPR | - | - | - | - | - | + | + | - | - | - |
| Q02790 | FKB4_HUMAN | FK506-binding protein 4 | 2 FKBP_C  3 TPR | - | + | - | - | + | + | + | - | + | - |
| **HSP20** |  |  |  |  |  |  |  |  |  |  |  |  |  |
| P04792 | HS27_HUMAN | Heat shock 27 kDa protein | HSP20 | - | + | + | - | + | + | - | - | + | + |
| **ERP29_C** | |  |  |  |  |  |  |  |  |  |  |  |  |
| P30040 | ER29_HUMAN | Endoplasmic reticulum protein ERp29 [Precursor] | ERP29_C | - | + | - | - | + | - | + | - | + | + |
| **KE2** |  |  |  |  |  |  |  |  |  |  |  |  |  |
| Q9UHV9 | PFD2_HUMAN | Prefoldin subunit 2 | KE2 | - | + | - | - | - | - | - | - | - | + |
| **Prefoldin** | |  |  |  |  |  |  |  |  |  |  |  |  |
| Q15765 | PFD3_HUMAN | Prefoldin subunit 3 | Prefoldin | - | - | - | - | - | - | - | - | - | + |
| Table 1. Continued | | |  |  |  |  |  |  |  |  |  |  |  |
| **Acc. No** | **Abbr. Name** | Protein Name | Domain | **Saos-2** | **SK-N-SH** | HCT 116 | **CaOv-3** | **A549** | **HL-60** | **A-375** | **A-673** | **MCF-7** | **Hela** |
| **DUF704** | |  |  |  |  |  |  |  |  |  |  |  |  |
| O95433 | AHA1_HUMAN | Activator of 90 kDa heat shock protein ATPase homolog 1 | DUF704 | - | + | - | - | - | - | - | - | - | + |
| BAG |  |  |  |  |  |  |  |  |  |  |  |  |  |
| O95816 | BAG2_HUMAN | BAG-family molecular chaperone regulator-2 | BAG | - | - | - | - | + | - | - | - | - | + |
| **GrpE** |  |  |  |  |  |  |  |  |  |  |  |  |  |
| Q9HAV7 | GRE1_HUMAN | GrpE protein homolog 1, mitochondrial [Precursor] | GrpE | - | - | - | - | - | - | - | + | - | - |
| **DcpS** |  |  |  |  |  |  |  |  |  |  |  |  |  |
| Q9Y2S5 | Q9Y2S5 | HSPC015 | DcpS | - | - | - | - | - | + | - | - | - | - |

^1^ **Saos-2 :** bone; osteosarcoma ^2^ **SK-N-SH :** bone marrow neuroblastoma

^3^ **HCT 116 :** colon; colorectal carcinmoma ^4^ **Caov-3 :** ovary; adenocarcinoma

^5^ **A549 :** lung; carcinoma ^6^ **HL-60 :** peripheral blood; promyeloblast; acute promyelocytic leukaemia

^7^ **A-375 :** skin; malignant melanoma ^8^ **A-673 :** Rhabdomyosarcoma

^9^ **MCF-7 :** breast cancer ^10^**Hela :** cervix carcinoma
