# Supplementary material for: Expressional patterns of chaperones in ten human tumor cell lines
Source: Proteome Sci. 2004 Dec 14;2:8. doi: 10.1186/1477-5956-2-8 (PMC543454; doi:10.1186/1477-5956-2-8)
Supplement: Additional File 2 — Table 1-1. Identified proteins in different normal cell lines: Kidney, Lymphocyte, Fibroblast [file 1477-5956-2-8-S2.doc]

**Table 1-1.** Identified proteins in different cell lines: Kidney, Lymphocyte, Fibroblast (+ : Detected, - : Not-Detected, Acc. No: Accession Number in Swissprot, Abbr. Name: Abbreviation Name)

| **Acc. No** | **Abbr. Name** | Protein Name | **Domain** | **Kidney**  **HK-2** | **Lymphocyte 3610** | **Fibroblast** Hs 545 SK |
| --- | --- | --- | --- | --- | --- | --- |
| HSP90 / HATPas_C | | |  |  |  |  |
| P07900 | HS9A_HUMAN | Heat shock protein HSP 90-alpha | HATPas_C / HSP90 | + | - | - |
| P08238 | HS9B_HUMAN | Heat shock protein HSP 90-beta | HATPas_C / HSP90 | + | + | + |
| Q12931 | TRAL_HUMAN | Heat shock protein 75 kDa, mitochondrial [Precursor] | HATPas_C / HSP90 | + | + | - |
| **HSP70** |  |  |  |  |  |  |
| Q92598 | H105_HUMAN | Heat-shock protein 105 kDa | HSP70 | + | - | - |
| P08107 | HS71_HUMAN | Heat shock 70 kDa protein 1 | HSP70 | + | + | + |
| P34932 | HS74_HUMAN | Heat shock 70 kDa protein 4 | HSP70 | + | - | - |
| P38646 | GR75_HUMAN | Stress-70 protein, mitochondrial [Precursor] | HSP70 | + | + | + |
| P11021 | GR78_HUMAN | 78 kDa glucose-regulated protein [Precursor] | HSP70 | + | + | + |
| P11142 | HS7C_ HUMAN | Heat shock cognate 71 kDa protein | HSP70 | + | + | + |
| O95757 | OS94_HUMAN | Osmotic stress protein 94 | HSP70 | + | - | - |
| **Cpn60_TCP1** | | |  |  |  |  |
| P10809 | CH60_HUMAN | 60 kDa heat shock protein, mitochondrial [Precursor] | Cpn60_TCP1 | + | + | + |
| P17987 | TCPA_HUMAN | T-complex protein 1, alpha subunit | Cpn60_TCP1 | + | + | + |
| P78371 | TCPB_HUMAN | T-complex protein 1, beta subunit | Cpn60_TCP1 | + | + | + |
| P49368 | TCPG_HUMAN | T-complex protein 1, gamma subunit | Cpn60_TCP1 | - | + | + |
| P48643 | TCPE_HUMAN | T-complex protein 1, epsilon subunit | Cpn60_TCP1 | + | + | - |
| Table 1-1. Continued | | |  |  |  |  |
| **Acc. No** | **Abbr. Name** | Protein Name | Domain | **Kidney**  **HK-2** | **Lymphocyte 3610** | **Fibroblast** Hs 545 SK |
| **Cpn60_TCP1 – continued** | | |  |  |  |  |
| P40227 | TCPZ_HUMAN | T-complex protein 1, zeta subunit | Cpn60_TCP1 | + | + | - |
| Q99832 | TCPH_HUMAN | T-complex protein 1, eta subunit | Cpn60_TCP1 | + | - | - |
| **Thioredoxin** | | |  |  |  |  |
| P07237 | PDI_HUMAN | Protein disulfide isomerase precursor | 2 Thioredoxin | + | + | + |
| P30101 | PDA3_HUMAN | Protein disulfide isomerase A3 [Precursor] | 2Thioredoxin | + | + | + |
| Q15084 | PDA6_HUMAN | Protein disulfide somerase A6 [Precursor] | 2 Thioredoxin | + | + | + |
| **TPR** |  |  |  |  |  |  |
| P31948 | IEFS_HUMAN | Stress-induced-phosphoprotein 1 | 9TPR | - | + | + |
| **HSP20** |  |  |  |  |  |  |
| P04792 | HS27_HUMAN | Heat shock 27 kDa protein | HSP20 | + | - | + |
