# Supplementary material for: Expressional patterns of chaperones in ten human tumor cell lines
Source: Proteome Sci. 2004 Dec 14;2:8. doi: 10.1186/1477-5956-2-8 (PMC543454; doi:10.1186/1477-5956-2-8)
Supplement: Additional File 3 — Table 2. Theoretical molecular weight, theoretical pI, observed pI, total score and peptide matched of molecular chaperones in tumor cell lines [file 1477-5956-2-8-S3.doc]

**Table 2**. Theoretical molecular weight, theoretical p*I* , observed p*I*, total score and peptide matched of molecular chaperones in tumor cell lines.

(Tr..Mr : Theoretical Molecular weights , Tr. p*I* : Theoretical Isoelectric point )

| **Accession Number** | **Abbreviation Name** | **Protein Name** | **Cell lines** | **Total score** | **Peptide Mached** | **Observed**  **p*I*(pH)** | |
| --- | --- | --- | --- | --- | --- | --- | --- |
| **P07900** | **HS9A_HUMAN** | Heat shock protein HSP 90-alpha **Tr. Mr (Da) = 84542 Tr. p*I* (pH) = 4,94** | HCT 116 | 100 | 17 | | 5.65 |
|  |  |  | A549 | 192 | 32 | | 5.12 |
|  |  |  | A-673 | 86 | 17 | | 5.77 |
|  |  |  | MCF-7 | 249 229  185 | 36  34  28 | | 5.60  5.25  5.35 |
|  |  |  | Hela | 150 | 27 | | 5.50 |
| **P08238** | **HS9B_HUMAN** | **Heat shock protein HSP 90-beta**  **Tr. Mr (Da) = 83133 Tr. p*I* (pH) = 4,97** | Saos-2 | 110 | 17 | | 5.58 |
|  |  |  | Sk_N_SH | 90 | 23 | | 5.18 |
|  |  |  | HCT 116 | 153 | 28 | | 5.40 |
|  |  |  | HL_60 | 107 | 20 | | 5.71 |
|  |  |  | A-375 | 184  167 | 29  28 | | 5.38  5.41 |
|  |  |  | Hela | 116 | 19 | | 5.65 |
| **P14625** | **ENPL_HUMAN** | Endoplasmin [Precursor] **Tr. Mr (Da) = 92468 Tr. p*I* (pH) = 4,76** | Saos-2 | 254 | 46 | | 5.10 |
|  |  |  | SK-N-SH | 296 68 | 41  20 | | 5.08  5.10 |
|  |  |  | HCT 116 | 68 | 21 | | 5.00 |
|  |  |  | A549 | 199 | 31 | | 5.00 |
|  |  |  | HL-60 | 106 262 | 21  43 | | 5.18  5.45 |
|  |  |  | A-375 | 323  146  203  134 | 49  30  38  27 | | 5.00  5.15  5.00  5.02 |
|  |  |  | A-673 | 69 | 17 | | 5.02 |
|  |  |  | MCF-7 | 74 161  282 | 15  29  44 | | 5.10  5.25  5.30 |
|  |  |  | Hela | 266 | 41 | | 4.90 |
| **Q12931** | **TRAL_HUMAN** | Heat shock protein 75 kDa, mitochondrial [Precursor] **Tr. Mr (Da) = 80010 Tr. p*I* (pH) = 8,05** | SK-N-SH | 120 | 33 | | 6.25 |
|  |  |  | CaOv-3 | 195 | 40 | | 6.20 |
|  |  |  | A549 | 253 | 47 | | 6.18 |
|  |  |  | A-375 | 236  295 | 43  44 | | 6.40  6.30 |
|  |  |  | A-673 | 121 | 25 | | 6.35 |
|  |  |  | MCF-7 | 294 | 43 | | 6.40 |
|  |  |  | Hela | 209 | 38 | | 6.50 |
| **Q9NTK6** | **Q9NTK6** | **88.1% homologous to isoform of HSP90-beta**  **Tr. Mr (Da) = 84843 Tr. p*I* (pH) = 5,26** | A-673 | 74 85 | 17  20 | | 5.18  5.55 |
| **Q96GW1** | **Q96GW1** | **Similar to tumor rejection antigen (gp96) 1**  **Tr. Mr (Da) = 35449 Tr. p*I* (pH) = 4,62** | Saos-2 | 69 | 15 | | 4.80 |
| **Q92598** | **H105_HUMAN** | **Heat-shock protein 105 kDa**  **Tr. Mr (Da) = 96864 Tr. p*I* (pH) = 5,28** | CaOv-3 | 101 | 21 | | 5.33 |
|  |  |  | A-673 | 77 | 18 | | 5.45 |
|  |  |  | MCF-7 | 142 | 25 | | 5.48 |
|  |  |  | Hela | 67 | 20 | | 5.43 |
| **P08107** | **HS71_HUMAN** | **Heat shock 70 kDa protein 1**  **Tr. Mr (Da) = 70052 Tr. p*I* (pH) = 5,48** | Saos-2 | 272 | 41 | | 5.55 |
|  |  |  | HCT 116 | 209 | 33 | | 5.60 |
|  |  |  | A549 | 105 | 22 | | 5.60 |
|  |  |  | HL-60 | 285 | 38 | | 5.60 |
|  |  |  | A-375 | 295 | 40 | | 5.60 |
|  |  |  | A-673 | 8498 | 17  19 | | 5.60  5.55 |
|  |  |  | MCF-7 | 95 | 19 | | 5.63 |
|  |  |  | Hela | 349 | 45 | | 5.60 |
| **P54652** | **HS72_HUMAN** | **Heat shock-related 70 kDa protein 2**  **Tr. Mr (Da) = 70020 Tr. p*I* (pH) = 5,56** | SK-N-SH | 82 | 21 | | 5.64 |
| **P34932** | **HS74_HUMAN** | **Heat shock 70 kDa protein 4**  **Tr. Mr (Da) = 94299 Tr. p*I* (pH) = 5,18** | SK-N-SH | 213 | 39 | | 5.25 |
|  |  |  | A549 | 149 | 32 | | 5.27 |
|  |  |  | HL-60 | 189 | 35 | | 5.25 |
|  |  |  | A-673 | 216 | 37 | | 5.25 |
| **P38646** | **GR75_HUMAN** | **Stress-70 protein, mitochondrial [Precursor]**  **Tr. Mr (Da) = 73680 Tr. p*I* (pH) = 5,87** | Saos-2 | 169 | 31 | | 5.60 |
|  |  |  | SK-N-SH | 170 153 | 26  32 | | 5.57  5.61 |
|  |  |  | HCT 116 | 174 256 | 36  41 | | 5.52  5.61 |
|  |  |  | CaOv-3 | 177 | 28 | | 5.50 |
|  |  |  | A549 | 278 | 43 | | 5.55 |
|  |  |  | HL-60 | 275 123  385  168 | 42  24  50  30 | | 5.60  5.65  5.60  5.55 |
|  |  |  | A-375 | 215 303  207  281  259  305 | 36  44  36  41  44  38 | | 5.65  5.55  5.55  5.62  5.45  5.62 |
|  |  |  | A-673 | 122 | 24 | | 5.67 |
| **P11021** | **GR78_HUMAN** | **78 kDa glucose-regulated protein [Precursor]**  **Tr. Mr (Da) = 72332 Tr. p*I* (pH) = 5,07** | Saos-2 | 189 | 28 | | 5.05 |
|  |  |  | SK-N-SH | 110 | 23 | | 5.20 |
|  |  |  | HCT 116 | 133 | 26 | | 5.05 |
|  |  |  | CaOv-3 | 232 | 41 | | 5.13 |
|  |  |  | A549 | 365 223 | 48  36 | | 5.15  5.10 |
|  |  |  | HL-60 | 342 328 | 31  43 | | 5.08  5.11 |
|  |  |  | A-375 | 389 350  368 | 49  47  46 | | 5.08  5.11  5.12 |
|  |  |  | A-673 | 325 226  304 | 45  32  43 | | 5.15  5.19  5.21 |
|  |  |  | Hela | 153 119  362 | 28  24  45 | | 5.50  4.90  5.10 |
| **Q9UK02** | **Q9UK02** | **BiP protein [Fragment]**  **Tr. Mr (Da) = 70931 Tr. p*I* (pH) = 5,23** | MCF-7 | 111  145 | 18  24 | | 5.3  5.3 |
| **P11142** | **HS7C_HUMAN** | **Heat shock cognate 71 kDa protein**  **Tr. Mr (Da) = 70898 Tr. p*I* (pH) = 5,37** | Saos-2 | 188 77  168 | 34  20  33 | | 5.42  6.10  5.50 |
|  |  |  | SK-N-SH | 144 | 30 | | 5.35 |
|  |  |  | HCT 116 | 204 | 38 | | 5.45 |
|  |  |  | CaOv-3 | 143 149  134 | 30  28  28 | | 5.51  5.28  5.38 |
|  |  |  | A549 | 195 132  88 | 26  28  16 | | 5.32  5.50  5.38 |
|  |  |  | HL-60 | 179 261  291  229 | 30  40  42  36 | | 5.38  5.45  5.50  5.55 |
|  |  |  | A-375 | 299 257  148  118  285 | 46  40  27  23  44 | | 5.48  5.43  6.10  5.28  5.38 |
|  |  |  | A-673 | 170 173  187  124 | 30  32  33  23 | | 5.95  5.65  5.40  5.58 |
|  |  |  | MCF-7 | 201  254 | 28  37 | | 5.56  5.48 |
|  |  |  | Hela | 85 158 | 21  27 | | 5.90  5.40 |
| **Q9Y4L1** | **OXRP_HUMAN** | **150 kDa oxygen-regulated protein [Precursor]**  **Tr. Mr (Da) = 111335 Tr. p*I* (pH) = 5,16** | MCF-7 | 236 312 | 37  43 | | 5.35  5.36 |
| **Q96IS6** | **Q96IS6** | **Similar to heat shock cognate 71-kd protein**  **Tr. Mr (Da) = 64602 Tr. p*I* (pH) = 5,36** | HL-60 | 77 | 13 | | 6.12 |
| **P10809** | **CH60_HUMAN** | **60 kDa heat shock protein, mitochondrial [Precursor]**  **Tr. Mr (Da) = 61054 Tr. p*I* (pH) = 5,70** | Saos-2 | 94  201  217 | 12  40  42 | | 5.42  5.35  5.28 |
|  |  |  | SK-N-SH | 109161 | 27  34 | | 5.48  5.35 |
|  |  |  | HCT 116 | 89 223  195  200 | 17  39  29  39 | | 5.28  5.45  5.40  5.32 |
|  |  |  | CaOv-3 | 229 | 35 | | 5.37 |
|  |  |  | A549 | 65  137 | 8  28 | | 5.35  5.63 |
|  |  |  | HL-60 | 159 421 | 28  48 | | 5.32  5.40 |
|  |  |  | A-375 | 355 123  383  459  459 | 49  24  49  49  57 | | 5.49  5.49  5.32  5.40  5.40 |
|  |  |  | A-673 | 203 62  311 | 29  13  44 | | 5.38  5.38  5.43 |
|  |  |  | Hela | 336 439  92 | 47  56  19 | | 5.30  5.40  5.25 |
| **P17987** | **TCPA_HUMAN** | **T-complex protein 1, alpha subunit**  **Tr. Mr (Da) = 60343 Tr. p*I* (pH) = 5,80** | Saos-2 | 218  79 | 40  14 | | 5.82  5.75 |
|  |  |  | SK-N-SH | 148 | 28 | | 5.83 |
|  |  |  | HCT 116 | 92 | 18 | | 5.82 |
|  |  |  | CaOv-3 | 207 | 34 | | 5.80 |
|  |  |  | A549 | 161 | 24 | | 5.72 |
|  |  |  | HL-60 | 292 | 44 | | 5.83 |
|  |  |  | A-375 | 422 | 52 | | 5.85 |
|  |  |  | MCF-7 | 233  206 | 31  31 | | 5.88  5.79 |
|  |  |  | Hela | 228 | 32 | | 5.85 |
| **P78371** | **TCPB_HUMAN** | **T-complex protein 1, beta subunit**  **Tr. Mr (Da) = 57488 Tr. p*I* (pH) =6,01** | Saos-2 | 69 | 21 | | 6.10 |
|  |  |  | SK-N-SH | 178 83 | 25  23 | | 6.00  6.10 |
|  |  |  | HCT 116 | 195 | 35 | | 6.12 |
|  |  |  | CaOv-3 | 148 | 28 | | 6.08 |
|  |  |  | A549 | 158  73 | 31  19 | | 6.02  6.02 |
|  |  |  | HL-60 | 87 | 24 | | 6.13 |
|  |  |  | A-375 | 217 | 35 | | 6.03 |
|  |  |  | A-673 | 194 | 32 | | 6.05 |
|  |  |  | Hela | 98 | 23 | | 6.15 |
| **P49368** | **TCPG_HUMAN** | **T-complex protein 1, gamma subunit**  **Tr. Mr (Da) = 60402 Tr. p*I* (pH) = 6,10** | Saos-2 | 110 | 21 | | 6.10 |
|  |  |  | SK-N-SH | 84 | 15 | | 6.10 |
|  |  |  | HCT 116 | 66 | 15 | | 6.12 |
|  |  |  | A549 | 257  170 | 34  30 | | 6.01  5.90 |
|  |  |  | HL-60 | 256 | 33 | | 6.15 |
|  |  |  | A-375 | 237 | 34 | | 6.12 |
|  |  |  | Hela | 106 | 20 | | 6.15 |
| **P48643** | **TCPE_HUMAN** | **T-complex protein 1, epsilon subunit**  **Tr. Mr (Da) = 59671 Tr. p*I* (pH) = 5,45** | Saos-2 | 156 | 30 | | 5.57 |
|  |  |  | HCT 116 | 107 | 20 | | 5.50 |
|  |  |  | A549 | 127  111 | 25  23 | | 5.40  5.48 |
|  |  |  | MCF-7 | 75 | 16 | | 5.91 |
|  |  |  | Hela | 204 | 35 | | 5.54 |
| **P40227** | **TCPZ_HUMAN** | **T-complex protein 1, zeta subunit**  **Tr. Mr (Da) = 58024 Tr. p*I* (pH) = 6,24** | Saos-2 | 160 | 32 | | 6.29 |
|  |  |  | SK-N-SH | 188 | 31 | | 6.30 |
|  |  |  | HCT 116 | 141 | 26 | | 6.37 |
|  |  |  | CaOv-3 | 90 | 15 | | 6.15 |
|  |  |  | A549 | 185 | 31 | | 6.20 |
|  |  |  | HL-60 | 238 | 35 | | 6.36 |
|  |  |  | A-375 | 253 | 37 | | 6.40 |
|  |  |  | A-673 | 181  198 | 31  33 | | 6.20  6.35 |
|  |  |  | MCF-7 | 235 | 38 | | 6.35 |
|  |  |  | Hela | 155  327 | 29  43 | | 6.30  6.40 |
| **Q9BU08** | **Q9BU08** | **96% homologous to isororm of T-complex protein 1, epsilon subunit**  **Tr. Mr (Da) = 59468 Tr. p*I* (pH) = 5,45** | SK-N-SH | 77  86  133 | 17  20  29 | | 5.79  5.64  5.60 |
|  |  |  | HCT 116 | 126 | 29 | | 5.60 |
|  |  |  | A549 | 215  233 | 37  38 | | 5.52  5.60 |
|  |  |  | HL-60 | 275  197 | 38  29 | | 5.60  5.65 |
|  |  |  | A-375 | 303 | 43 | | 5.60 |
|  |  |  | A-673 | 117 | 21 | | 5.60 |
|  |  |  | MCF-7 | 271 | 36 | | 5.63 |
| **P31689** | **DJA1_HUMAN** | **DnaJ homolog subfamily A member1**  **Tr. Mr (Da) = 44868 Tr. p*I* (pH) = 6,65** | A549 | 108 | 20 | | 6.80 |
| **O60884** | **DJA2_HUMAN** | **DnaJ homolog subfamily A member 2**  **Tr. Mr (Da) = 45745 Tr. p*I* (pH) = 6,06** | SK-N-SH | 80 | 16 | | 6.05 |
| **Q9UBS4** | **DJBB_HUMAN** | **DnaJ homolog subfamily B member 11 [Precursor]**  **Tr. Mr (Da) = 40513 Tr. p*I* (pH) = 5,81** | SK-N-SH | 85 | 16 | | 6.00 |
|  |  |  | A549 | 97 | 14 | | 6.02 |
|  |  |  | Hela | 93 | 16 | | 6.05 |
| **P07237** | **PDI_HUMAN** | Protein disulfide isomerase[precursor] **Tr. Mr (Da) = 57116 Tr. p*I* (pH) = 4.76** | CaOva3 | 127 | 20 | | 4.94 |
|  |  |  | A549 | 95 | 8 | | 4.99 |
|  |  |  | HL-60 | 146  83 | 21  14 | | 4.90  4.90 |
| **P30101** | **PDA3_HUMAN** | **Protein disulfide isomerase A3 [Precursor]**  **Tr. Mr (Da) = 56782 Tr. p*I* (pH) = 5,98** | Saos-2 | 112  321 | 17  44 | | 5.73  5.63 |
|  |  |  | SK-N-SH | 122  112  103 | 18  24  21 | | 5.68  5.73  5.78 |
|  |  |  | HCT 116 | 132  189 | 24  32 | | 5.78  5.73 |
|  |  |  | CaOv-3 | 158  120  146 | 28  24  23 | | 5.77  5.72  5.66 |
|  |  |  | A549 | 321 | 44 | | 5.70 |
|  |  |  | HL-60 | 339  197 | 41  25 | | 5.74  5.74 |
|  |  |  | A-375 | 219  233  293 | 34  35  37 | | 5.70  5.78  5.73 |
|  |  |  | A-673 | 169 | 25 | | 5.80 |
|  |  |  | MCF-7 | 185 | 26 | | 5.80 |
| **Q15084** | **PDA6_HUMAN** | **Protein disulfide isomerase-related protein 5**  **Tr. Mr (Da) = 48121 Tr. p*I* (pH) = 4,95** | Saos-2 | 69 | 14 | | 5.15 |
|  |  |  | A549 | 131 | 20 | | 5.20 |
|  |  |  | HL-60 | 121 | 19 | | 5.25 |
|  |  |  | A-375 | 226  88 | 29  15 | | 5.20  5.25 |
|  |  |  | A-673 | 130  93 | 20  15 | | 5.25  5.22 |
|  |  |  | Hela | 217 | 28 | | 5.18 |
| **P05092** | **PPIA_HUMAN** | **Peptidyl-prolyl cis-trans isomerase A**  **Tr. Mr (Da) = 17881 Tr. p*I* (pH) = 7,82** | SK-N-SH | 63 | 11 | | 6.55 |
|  |  |  | A549 | 102  112 | 17  16 | | 6.78  6.35 |
|  |  |  | HL-60 | 137 | 20 | | 6.35 |
|  |  |  | A-375 | 67 | 10 | | 5.25 |
|  |  |  | A-673 | 68  143 | 10  18 | | 6.77  7.70 |
| **P31948** | **IEFS_HUMAN** | **Stress-induced-phosphoprotein 1**  **Tr. Mr (Da) = 62639 Tr. p*I* (pH) = 6,40** | SK-N-SH | 93  206 | 20  36 | | 6.00  6.57 |
|  |  |  | HCT 116 | 253 | 38 | | 6.30 |
|  |  |  | CaOv-3 | 150 | 27 | | 6.34 |
|  |  |  | A549 | 180  455 | 29  55 | | 6.52  6.38 |
|  |  |  | HL-60 | 185 | 26 | | 6.70 |
|  |  |  | A-375 | 423  347 | 51  41 | | 6.60  6.40 |
|  |  |  | A-673 | 114  338  198 | 20  50  30 | | 6.20  6.35  6.65 |
|  |  |  | MCF-7 | 391  415  135 | 47  50  24 | | 6.35  6.50  6.65 |
|  |  |  | Hela | 444  344  143 | 53  49  25 | | 6.40  6.70  6.30 |
| **P50502** | **PPIA_HUMAN** | **Hsc70-interacting protein**  **Tr. Mr (Da) = 41331 Tr. p*I* (pH) = 5,18** | HL-60 | 68 | 11 | | 5.15 |
|  |  |  | A-375 | 93 | 18 | | 5.19 |
| **Q02790** | **FKB4_HUMAN** | **FK506-binding protein 4**  **Tr. Mr (Da) = 51673 Tr. p*I* (pH) = 5,35** | SK-N-SH | 87 | 15 | | 5.53 |
|  |  |  | A549 | 197 | 27 | | 5.40 |
|  |  |  | HL-60 | 171 | 27 | | 5.55 |
|  |  |  | A-375 | 93  197 | 19  31 | | 5.51  5.48 |
|  |  |  | MCF-7 | 127 | 21 | | 5.62 |
| **P04792** | **HS27_HUMAN** | **Heat shock 27 kDa protein**  **Tr. Mr (Da) = 22782 Tr. p*I* (pH) = 5,98** | SK-N-SH | 87 | 13 | | 5.89 |
|  |  |  | HCT 116 | 68 | 8 | | 5.65 |
|  |  |  | A549 | 130 | 14 | | 5.60 |
|  |  |  | HL-60 | 91 | 13 | | 5.95 |
|  |  |  | MCF-7 | 197  173  201  201 | 21  20  22  22 | | 5.76  5.71  5.95  5.70 |
|  |  |  | Hela | 160  168 | 18  18 | | 5.65  6.00 |
|  |  |  |  |  |  |  |  |
| **P30040** | **ER29_HUMAN** | **Endoplasmic reticulum protein ERp29 [Precursor]**  **Tr. Mr (Da) = 28993 Tr. p*I* (pH) = 6,77** | SK-N-SH | 107 | 17 | | 6.08 |
|  |  |  | A549 | 135  82 | 17  12 | | 5.94  5.70 |
|  |  |  | A-375 | 120  115 | 19  16 | | 5.75  6.10 |
|  |  |  | MCF-7 | 153 | 18 | | 6.15 |
|  |  |  | Hela | 249 | 30 | | 6.10 |
| **Q9UHV9** | **PFD2_HUMAN** | **Prefoldin subunit 2**  **Tr. Mr (Da) = 16647 Tr. p*I* (pH) = 6,20** | SK-N-SH | 68 | 8 | | 6.00 |
|  |  |  | Hela | 165 | 16 | | 6.05 |
| **Q15765** | **PFD3_HUMAN** | **Prefoldin subunit 3**  **Tr. Mr (Da) = 21448 Tr. p*I* (pH) = 6,63** | Hela | 181 | 21 | | 6.20 |
| **O95433** | **AHA1_HUMAN** | **Activator of 90 kDa heat shock protein ATPase homolog 1**  **Tr. Mr (Da) = 38274 Tr. p*I* (pH) = 5,41** | SK-N-SH | 86 | 17 | | 5.52 |
|  |  |  | Hela | 75 | 12 | | 5.54 |
| **O95816** | **BAG2_HUMAN** | **BAG-family molecular chaperone regulator-2**  **Tr. Mr (Da) = 23771 Tr. p*I* (pH) = 6,25** | A549 | 66 | 9 | | 6.10 |
|  |  |  | Hela | 78 | 10 | | 5.85 |
| **Q9HAV7** | **GRE1_HUMAN** | **GrpE protein homolog 1, mitochondrial [Precursor]**  **Tr. Mr (Da) = 24279 Tr. p*I* (pH) = 8,24** | A-673 | 166 | 20 | | 5.90 |
| **Q9Y2S5** | **Q9Y2S5** | **HSPC015**  **Tr. Mr (Da) = 38608 Tr. p*I* (pH) = 5,93** | HL-60 | 179 | 22 | | 5.90 |
