# Supplementary material for: Expressional patterns of chaperones in ten human tumor cell lines
Source: Proteome Sci. 2004 Dec 14;2:8. doi: 10.1186/1477-5956-2-8 (PMC543454; doi:10.1186/1477-5956-2-8)
Supplement: Additional File 4 — Table 3. The list of tumor cell lines [file 1477-5956-2-8-S4.doc]

#### Table 3. The list of cell line

| **ATCC Number** | **Designation** | Tissue |
| --- | --- | --- |
| HTB-85 | Saos-2 | bone; osteosarcoma |
| HTB-75 | CaOv-3 | ovary; adenocarcinoma |
| HTB-11 | SK-N-SH | brain; metastatic site: bone marrow neuroblastoma |
| CCL-247 | HCT116 | colon; colorectal carcinoma |
| CCL-185 | A549 | lung; carcinoma |
| CCL-240 | HL-60 | peripheral blood; promyeloblast; acute promyelocytic leukaemia |
| CRL-1619 | A-375 | skin; malignant melanoma |
| CRL-1598 | A-673 | muscle; rhabdomyosarcoma |
| HTB-22 | MCF-7 | mammary gland; breast; epithelial; metastatic site:  pleural effusion adenocarcinoma |
| CCL-2 | Hela | cervix; epithelial; adenocarcinoma |
